# Emotional intelligence and loneliness in eating disorders: a cluster-analytic study across diagnostic categories

**DOI:** 10.1186/s40337-025-01411-x

**Published:** 2025-10-10

**Authors:** Paolo Meneguzzo, Anna Marzotto, Fabio Conti, Barbara Mezzani, Luca Maggi, Patrizia Todisco

**Affiliations:** 1 Department of Neuroscience, University of Padova, Via Giustiniani 2, Padova, 35128 Italy; 2 Padova Neuroscience Center, University of Padova, Padova, Italy; 3 Eating Disorder Unit, Casa di Cura Villa Margherita-KOS Group, Arcugnano, Vicenza Italy; 4 Psycho-Nutritional Center, Verona, Vicenza, Italy; 5 Eating Disorders Unit, Casa di Cura Villa dei Pini-KOS Group, Firenze, Italy; 6 Eating Disorders Unit, Casa di Cura Villa Armonia-KOS Group, Roma, Italy; 7 Eating Disorders Unit, Casa di Cura Ville di Nozzano-KOS Group, Nozzano, Lucca, Italy

**Keywords:** Eating disorders, Emotional intelligence, Loneliness, Cluster analysis, Transdiagnostic, Emotion regulation

## Abstract

**Objective:**

The present study aimed to investigate emotional intelligence and loneliness in individuals with eating disorders (EDs) using a transdiagnostic approach. Specifically, it sought to identify emotional-loneliness profiles through cluster analysis and evaluate their association with clinical characteristics and diagnostic categories.

**Method:**

A total of 371 participants (220 with EDs and 151 healthy controls) completed self-report measures including the Wong and Law Emotional Intelligence Scale (WLEIS), the UCLA Loneliness Scale, and the Eating Disorder Examination Questionnaire (EDE-Q). K-means cluster analysis was performed on standardized WLEIS and UCLA scores. Between-group comparisons and post hoc tests were conducted to assess differences across clusters in ED severity, BMI, age, and diagnosis. Logistic and chi-square analyses explored diagnostic distribution and predictive associations.

**Results:**

Three distinct clusters emerged: (1) Low Emotional Intelligence/High Loneliness (*n* = 130), (2) Moderate EI/Moderate Loneliness (*n* = 141), and (3) High EI/Low Loneliness (*n* = 100). Cluster 1 showed the most adaptive profile, while Cluster 0 exhibited the highest ED severity and loneliness. Diagnostic category distribution differed significantly across clusters (χ²(8) = 89.56, *p* < .001), but emotional profiles did not align exclusively with specific diagnoses, supporting a transdiagnostic model. Emotional intelligence and loneliness significantly predicted ED status.

**Conclusion:**

Emotional intelligence and loneliness form meaningful psychological profiles that transcend ED diagnoses and are associated with clinical severity. Assessing these factors may enhance early detection and inform targeted interventions. Future studies should explore the role of early adversity and trauma in shaping these profiles.

## Plain language summary

People with eating disorders often struggle not only with food and body image, but also with understanding and managing their emotions and feeling connected to others. In this study, we explored whether emotional intelligence (how well someone can understand and manage emotions) and loneliness might help us better understand different types of people who experience eating disorders. We asked over 370 individuals—including people with different eating disorder diagnoses and people without eating disorders—to complete questionnaires about emotions, loneliness, and eating behaviors. We then used a statistical method to group people based on their emotional profiles, not their diagnosis. We found three main groups: one with low emotional skills and high loneliness, one with average emotional skills and loneliness, and one with high emotional skills and low loneliness. The group with the lowest emotional intelligence and highest loneliness showed the most severe eating disorder symptoms. These emotional profiles did not match specific diagnoses, suggesting that traditional labels (like anorexia nervosa or bulimia) may not fully capture the emotional struggles of individuals. Understanding emotional patterns—beyond diagnosis—can help professionals offer more personalized support and may improve early identification and treatment of eating disorders.

## Background

Eating disorders (EDs) are complex psychiatric conditions that are often marked by disordered eating behaviors, body image disturbances, and significant psychological distress [1]. Although diagnostic systems like the DSM-5-TR define discrete categories such as anorexia nervosa, bulimia nervosa, and binge-eating disorder, growing evidence suggests that EDs may be better conceptualized along dimensional and transdiagnostic lines, reflecting shared psychological traits across diagnostic boundaries [2–4].

One key transdiagnostic feature in EDs is difficulty with emotional functioning, particularly in understanding, expressing, and regulating emotional experiences [5–7]. These impairments are associated with the development and maintenance of eating disorder behaviors and have been linked to poorer treatment outcomes [8–10]. A widely used construct that captures these emotional processes is emotional intelligence (EI), defined as the capacity to perceive, understand, use, and regulate emotions in oneself and others [11, 12]. EI is a multidimensional construct typically encompassing four key components: self-emotion appraisal, which refers to the ability to understand and interpret one’s own emotional states; others’ emotion appraisal, or the capacity to accurately perceive and interpret others’ emotions; use of emotion, meaning the ability to harness emotions to guide thinking and motivation; and regulation of emotion, which involves effectively managing emotional responses.

Individuals with EDs frequently show impairments across these domains, particularly in recognizing and regulating their own emotions [13–15]. Such difficulties may limit their ability to respond adaptively to distress and contribute to the use of maladaptive coping strategies, such as restrictive eating, purging, or bingeing, to modulate internal states. Low emotional intelligence has also been linked to impaired interpersonal functioning, emotional avoidance, and a limited capacity for affective communication—factors that may further perpetuate disordered eating behaviors [16–18].

Closely related to these emotional difficulties is the experience of loneliness, defined as the distressing subjective sense of social disconnection or lack of meaningful interpersonal relationships [19, 20]. Loneliness has been increasingly recognized as a transdiagnostic vulnerability factor in EDs and other mental health conditions. Research suggests that individuals with EDs often report high levels of loneliness, regardless of objective social support, and that loneliness may both precede and follow the onset of eating disorder symptoms [21–24]. This bidirectional relationship may contribute to a vicious cycle of social withdrawal, emotional dysregulation, and symptom exacerbation. Moreover, interpersonal difficulties commonly reported by individuals with EDs—such as mistrust, fear of intimacy, or avoidance of support—may reflect deeper emotional processing impairments that contribute to both loneliness and eating pathology [13, 25, 26].

Although the individual roles of emotional intelligence and loneliness in EDs are increasingly well-documented, few studies have examined how these traits co-occur or interact within individuals. A person-centered analytic approach, such as cluster analysis, allows for the identification of naturally occurring emotional–interpersonal profiles that may cut across traditional diagnostic categories [27]. These profiles may offer insight into affective phenotypes that underlie distinct patterns of ED vulnerability, chronicity, and severity. Identifying such emotional profiles could also enhance treatment personalization, for example by highlighting the need for emotion-focused or interpersonal interventions in subgroups of patients with pronounced emotional impairments.

The present study aimed to identify distinct profiles of emotional intelligence and loneliness in a large transdiagnostic sample of individuals with and without eating disorders. Using cluster analysis, we sought to determine whether naturally occurring emotional profiles were associated with differences in eating disorder symptoms, illness duration, and body mass index (BMI). We hypothesized that a maladaptive emotional profile—characterized by low emotional intelligence (particularly self-emotion appraisal and emotion regulation) and high loneliness—would be associated with greater eating disorder severity, regardless of diagnostic status. This would support the idea that emotional and interpersonal difficulties represent transdiagnostic, trait-like vulnerabilities, rather than illness-specific or state-dependent markers.

## Methods

### Participants

The final sample consisted of 371 cisgender women of White Italian background, aged between 13 and 73 years (M = 26.27, SD = 9.91), including 220 individuals with a diagnosed eating disorder (ED) and 151 women from general population referred as healthy controls (HC). Information on sexual orientation was not collected. The clinical group encompassed a range of DSM-5 diagnoses: anorexia nervosa restricting type (ANr, *n* = 95), anorexia nervosa binge–purge type (ANbp, *n* = 34), bulimia nervosa (BN, *n* = 36), binge eating disorder (BED, *n* = 43), and other specified feeding or eating disorders (OSFED, *n* = 26). Diagnoses were made through the same structured clinical interview conducted by experienced clinicians in specialized ED centers [28].

Control participants were recruited from the community and screened to ensure the absence of current or past EDs or major psychiatric disorders. Exclusion criteria for all participants included psychotic symptoms, acute manic episodes, and major neurocognitive impairment.

Participants provided informed consent before participation. The study protocol was approved by the Vicenza Ethics Committee (67/21), and all procedures conformed to the principles of the Declaration of Helsinki.

### Measures

All participants completed a comprehensive battery of standardized self-report questionnaires to assess emotional functioning, perceived social isolation, and eating disorder psychopathology. Emotional intelligence was measured using the Wong and Law Emotional Intelligence Scale (WLEIS), a 16-item instrument rated on a 7-point Likert scale [29]. The WLEIS evaluates four distinct domains of emotional intelligence: Self-Emotion Appraisal, Others’ Emotion Appraisal, Use of Emotion, and Regulation of Emotion. Higher scores indicate stronger emotional competencies in each domain.

Perceived loneliness was assessed using the UCLA Loneliness Scale, a 20-item measure widely used to evaluate subjective feelings of social isolation and lack of connectedness [30]. Respondents rate each item on a 4-point Likert scale, with higher scores reflecting greater loneliness.

To evaluate the severity of eating disorder psychopathology, participants completed the Eating Disorder Examination Questionnaire (EDE-Q) [31]. This 28-item instrument assesses both behavioral symptoms and cognitive features related to eating disorders over the past 28 days. It yields a global score as well as four subscale scores: Restraint, Eating Concern, Shape Concern, and Weight Concern.

In addition to self-report measures, clinical and demographic information was collected, including age, BMI, duration of illness, and number of hospitalizations. These variables were included in subsequent analyses to further contextualize the psychological profiles of the participants.

### Statistical analysis

All statistical analyses were conducted using JASP (version 0.19.3). Descriptive statistics were calculated for all demographic and clinical variables. To examine emotional functioning profiles, a K-means cluster analysis was performed using standardized scores from the four WLEIS subscales (Self-Emotion Appraisal, Others’ Emotion Appraisal, Use of Emotion, Regulation of Emotion) and total UCLA Loneliness Scale score. The optimal number of clusters was determined via silhouette coefficient values and theoretical interpretability, and validated using principal component analysis (PCA).

To assess group differences across clusters, Kruskal–Wallis H tests were used for continuous variables due to non-normal distributions and heterogeneity of variances. Post hoc pairwise comparisons were conducted using the Dunn–Bonferroni correction for multiple comparisons. For categorical variables, such as diagnostic category, chi-square tests of independence were used, followed by adjusted standardized residuals for post hoc interpretation of significant cell contributions.

Additionally, partial correlations controlling for age and BMI were used to examine the associations between emotional intelligence, loneliness, and eating disorder severity. A hierarchical logistic regression was conducted to evaluate whether emotional intelligence and loneliness predicted group membership (ED vs. HC), beyond demographic variables. The threshold for statistical significance was set at *p* <.05 for all analyses. Effect sizes were reported to provide estimates of practical significance.

## Results

Descriptive statistics and group comparisons for the clinical and emotional variables are presented in Table 1. Individuals with ED reported significantly higher levels of loneliness and lower emotional intelligence across all WLEIS subscales compared to HC. No significant differences emerged for age or BMI. Duration of illness and number of hospitalizations were specific to the ED group and not compared with controls.

**Table 1:** Descriptive statistics and group comparisons between eating disorder (ED) and healthy control (HC) groups

Variable	ED Mean (SD)	HC Mean (SD)	t	*p*	Cohen’s |d|
Age (years)	26.54 (11.79)	25.88 (6.24)	0.63	0.532	0.07
BMI	22.18 (11.70)	21.84 (2.75)	0.35	0.725	0.04
Loneliness (UCLA-LS)	48.98 (12.43)	36.05 (10.29)	10.54	< 0.001	1.12
Self-emotion appraisal	3.65 (1.45)	4.88 (1.17)	− 8.71	< 0.001	0.92
Others’ emotion appraisal	4.87 (1.38)	5.31 (1.24)	− 3.11	0.002	0.33
Use of emotion	3.46 (1.55)	4.61 (1.66)	− 6.85	< 0.001	0.72
Regulation of emotion	3.10 (1.54)	4.46 (1.55)	− 8.32	< 0.001	0.88
EDE-Q total score	4.04 (1.34)	1.48 (1.29)	18.35	< 0.001	1.94
Illness duration (years)	6.99 (8.16)	–	–	–	–
No. of hospitalizations	1.42 (2.65)	–	–	–	–

*BMI* Body Mass Index, *UCLA-LS* University of California, Los Angeles Loneliness Scale, *WLEIS* Wong and Law Emotional Intelligence Scale, *t*-values refer to independent samples *t*-tests between ED and HC groups. Negative *t* and *d* values indicate lower scores in the ED group

### Partial correlations between emotional intelligence, loneliness, and eating disorder severity

Partial correlations controlling for age and BMI revealed significant negative associations between emotional intelligence and both loneliness and eating disorder severity (see Table 2). Self-emotion appraisal was strongly and significantly associated with lower levels of loneliness (*r* = –.55, *p* <.001) and reduced eating disorder severity (*r* = –.49, *p* <.001). Use of emotion and regulation of emotion also showed significant negative correlations with loneliness (*r* = –.45 and –0.39, respectively; both *p* <.001) and eating disorder severity (*r* = –.38 and –0.34, respectively; both *p* <.001). Others’ emotion appraisal was moderately related to loneliness (*r* = –.34, *p* <.001), but only weakly to eating disorder severity (*r* = –.18, *p* <.001). These findings underscore the role of emotional self-awareness and regulation as protective factors in the emotional and clinical profiles of individuals with eating disorders.

**Table 2:** Partial correlations between emotional intelligence (WLEIS Subscale), loneliness, and eating disorder severity

	UCLA loneliness *r* (*p*)	EDE-Q global *r* (*p*)
Self-emotion appraisal	–0.55 (*p* <.001)	–0.49 (*p* <.001)
Others’ emotion appraisal	–0.34 (*p* <.001)	–0.18 (*p* <.001)
Use of emotion	–0.45 (*p* <.001)	–0.39 (*p* <.001)
Regulation of emotion	–0.48 (*p* <.001)	–0.45 (*p* <.001)

All values represent partial Pearson correlations (*r*), controlling for age and BMI. Higher WLEIS scores reflect greater emotional intelligence

### Logistic regression predicting clinical status

To explore whether emotional intelligence and loneliness could distinguish between clinical and control groups, a hierarchical logistic regression analysis was conducted. In Step 1, loneliness (UCLA), age, and BMI were entered as predictors. In Step 2, the four subscales of emotional intelligence (WLEIS) were added. The final model significantly improved model fit, χ²(4) = 26.95, *p* <.001, indicating that emotional intelligence contributed meaningful explanatory power beyond loneliness and demographic variables.

As shown in Table 3, lower scores on self-emotion appraisal (*B* = − 0.58, *p* =.014) and higher scores on others’ emotion appraisal (*B* = 0.28, *p* =.027) were significant predictors of group membership, with emotional intelligence components distinguishing individuals with eating disorders from healthy controls. Loneliness remained a strong predictor in the full model (*B* = 0.07, *p* <.001), underscoring the combined role of emotional and social factors in differentiating clinical and non-clinical participants.

**Table 3:** Logistic regression predicting ED group status from emotional intelligence and loneliness

Predictor	B	SE	Wald	*p*	Exp(B)
UCLA loneliness	0.07	0.01	35.40	< 0.001	1.08
Self-emotion appraisal	−0.58	0.24	6.03	0.014	0.56
Others’ emotion appraisal	0.28	0.13	4.89	0.027	1.32
Use of emotion	0.10	0.12	0.65	0.419	1.10
Regulation of emotion	−0.14	0.13	1.14	0.286	0.08
Age	0.03	0.02	3.65	0.056	1.03
BMI	0.01	0.02	0.71	0.400	1.01

B = unstandardized regression coefficient; SE = standard error; Exp(B) = odds ratio

### Cluster analysis and clinical characteristics

To identify distinct emotional and social profiles, a k-means cluster analysis was conducted on five standardized variables: self-emotion appraisal, others’ emotion appraisal, use of emotion, regulation of emotion (WLEIS subscales), and UCLA-Loneliness scale. The optimal three-cluster solution was selected based on theoretical interpretability and visual inspection using PCA. The resulting clusters differed meaningfully in emotional intelligence and loneliness levels, as well as clinical characteristics.

Cluster 0 (Low Emotional Intelligence/High Loneliness, *n* = 130) was characterized by significantly lower scores across all emotional intelligence domains and the highest levels of loneliness. This cluster also reported the highest eating disorder severity. The majority of participants were individuals with eating disorders (85.4%), especially those diagnosed with restricting-type Anorexia Nervosa (ANr, *n* = 47), followed by ANbp (*n* = 19), BN (*n* = 20), BED (*n* = 18), and OSFED (*n* = 8). Compared to other clusters, this group had a lower BMI and older age.

Cluster 1 (Moderate Emotional Intelligence/Moderate Loneliness, *n* = 141) exhibited intermediate levels of emotional intelligence and moderate levels of loneliness. This group included both HC (*n* = 58; 41.1%) and individuals with eating disorders (*n* = 83; 58.9%), with ANr (*n* = 32), ANbp (*n* = 13), BN (*n* = 15), BED (*n* = 18), and OSFED (*n* = 9). Participants in this cluster reported lower eating disorder severity compared to Cluster 0, and higher BMI.

Cluster 2 (High Emotional Intelligence/Low Loneliness, *n* = 100) demonstrated the most adaptive emotional profile, with the highest scores in all emotional intelligence subscales and the lowest levels of loneliness. This group had the lowest eating disorder severity, highest BMI, and was composed primarily of HC (74.0%). ED participants represented only 26.0% of this cluster, with ANr (*n* = 16), ANbp (*n* = 2), BN (*n* = 1), BED (*n* = 7), and OSFED (*n* = 9).

A chi-square test revealed a significant difference in diagnostic category distribution across clusters, χ²(8) = 89.56, *p* <.001. Post hoc analysis of adjusted standardized residuals indicated that Cluster 0 was significantly overrepresented by individuals with ANr (z = 4.79, *p* <.001), Cluster 1 showed no marked overrepresentation for any specific diagnosis, while Cluster 2 was significantly overrepresented by HC (z = 6.18, *p* <.001) and underrepresented by ANbp and BN diagnoses (both *p* <.01). The effect size, as measured by Cramér’s V, was **0.37**, indicating a moderate association between cluster membership and diagnostic category.

**Fig. 1:**
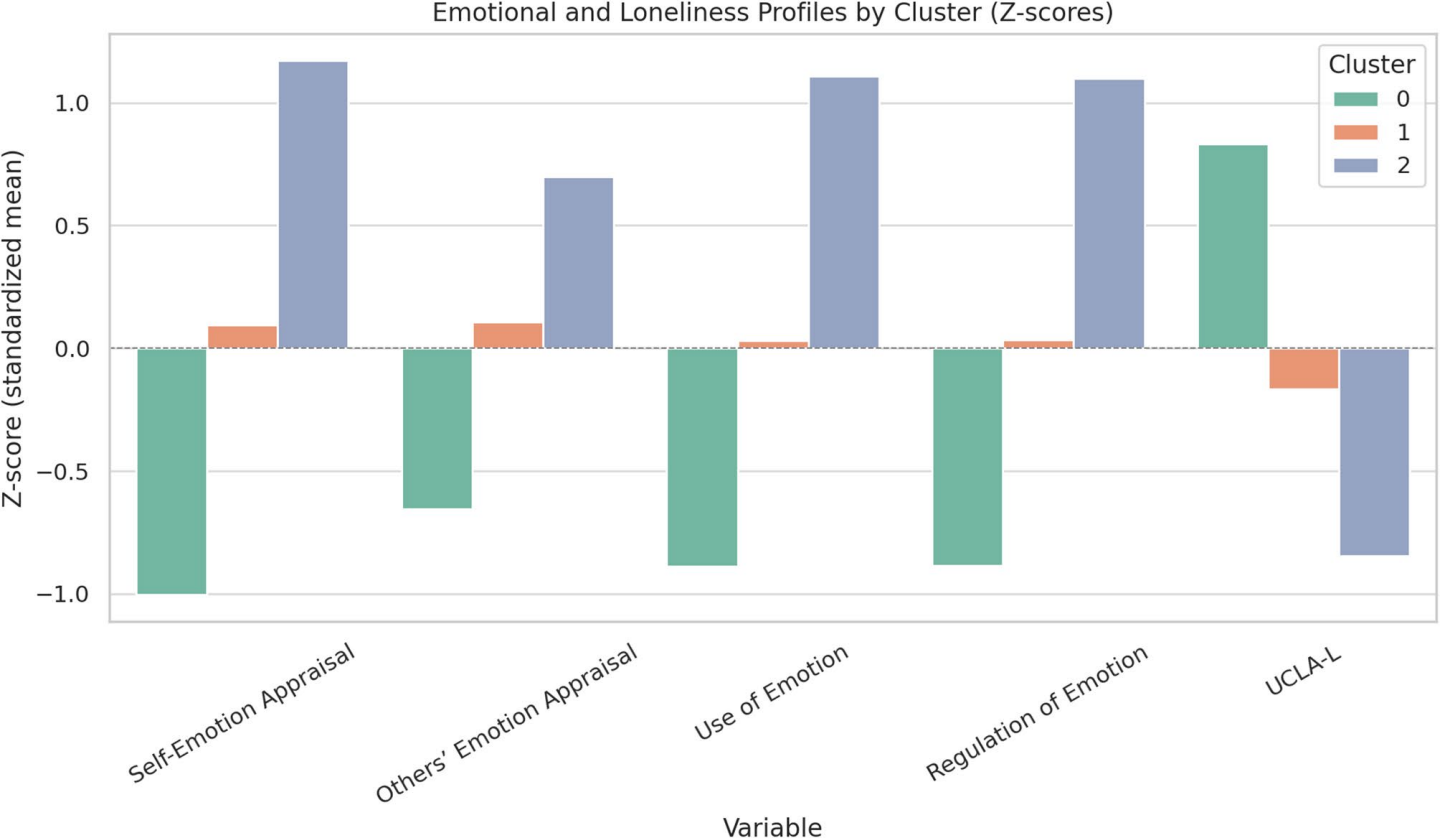
Emotional and loneliness profiles by cluster (Z-scores). Standardized mean scores (z-scores) for emotional intelligence subscales (Self-Emotion Appraisal, Others’ Emotion Appraisal, Use of Emotion, and Regulation of Emotion) and loneliness (UCLA-L) across the three identified clusters. Cluster 0 reflects a maladaptive profile characterized by low emotional intelligence and high loneliness. Cluster 1 shows intermediate levels across variables, while Cluster 2 represents an adaptive profile with high emotional intelligence and low loneliness. Error bars represent standard errors of the mean

To further explore whether clinical severity markers were associated with emotional-loneliness profiles, we compared illness duration and number of hospitalizations across the three clusters within the eating disorder group. Results indicated no statistically significant differences in illness duration across clusters, nor in the number of hospitalizations, χ²(2) = 2.47, *p* =.291. Details of clinical characteristics of the clusters are reported in Fig. 1; Table 4.

**Table 4:** Descriptive statistics and Kruskal-Wallis tests by cluster

Variable	Cluster 0 (*n* = 130)	Cluster 1 (*n* = 141)	Cluster 2 (*n* = 100)	H	*p*-value	Post hoc
WLEIS self-emotion appraisal	2.68 ± 0.92	4.29 ± 0.75	5.87 ± 0.54	282.83	< 0.001	2 > 0 (< 0.001) 2 > 1 (< 0.001) 1 > 0 (< 0.001)
WLEIS others-emotion appraisal	4.18 ± 1.42	5.19 ± 1.05	5.98 ± 0.77	110.40	< 0.001	2 > 0 (< 0.001) 2 > 1 (< 0.001) 1 > 0 (< 0.001)
WLEIS use of emotion	2.42 ± 1.04	3.98 ± 1.16	5.80 ± 0.93	226.24	< 0.001	2 > 0 (< 0.001) 2 > 1 (< 0.001) 1 > 0 (< 0.001)
WLEIS regulation of emotion	2.17 ± 1.04	3.71 ± 1.18	5.50 ± 0.91	221.12	< 0.001	2 > 0 (< 0.001) 2 > 1 (< 0.001) 1 > 0 (< 0.001)
UCLA-L	54.70 ± 10.64	41.51 ± 10.35	32.55 ± 7.93	165.17	< 0.001	2 > 0 (< 0.001) 2 > 1 (< 0.001) 1 > 0 (< 0.001)
EDE-Q global	3.27 ± 1.09	2.59 ± 1.07	1.39 ± 0.96	99.12	< 0.001	0 > 1 (< 0.001) 0 > 2 (< 0.001) 1 > 2 (< 0.001)
Age	27.31 ± 8.10	26.15 ± 7.51	25.10 ± 5.69	13.24	0.001	0 > 2 (0.001)
BMI	17.72 ± 2.20	19.04 ± 2.70	21.25 ± 3.63	8.011	0.018	2 > 0 (0.019)
Illness duration	7.05 ± 8.98	7.39 ± 7.75	5.50 ± 5.46	2.09	0.352	

Means (± standard deviations) of emotional intelligence subscales (WLEIS), loneliness (UCLA-L), eating disorder severity (EDE-Q global), age, BMI, and illness duration are presented for each cluster

## Discussion

This study aimed to identify transdiagnostic emotional profiles based on emotional intelligence and loneliness in individuals with and without EDs. The clustering solution revealed three distinct emotional-loneliness configurations that transcended traditional diagnostic boundaries, suggesting that affective functioning may represent a core dimension in understanding ED psychopathology.

The most maladaptive profile, characterized by low emotional intelligence—especially in self-emotion appraisal and emotion regulation—and high levels of loneliness, was associated with more severe ED symptoms, lower BMI, and a higher prevalence of restrictive and bulimic presentations. This pattern aligns with a body of literature suggesting that emotional unawareness and dysregulation are central features of EDs, particularly anorexia nervosa and bulimia nervosa [8, 32, 33]. These emotional deficits are thought to limit access to internal cues and promote the use of eating behaviors as strategies for affect regulation [34]. Social isolation, in turn, may be both a vulnerability factor and a consequence of eating disorder symptoms. Individuals often avoid social contexts to prevent eating in public, withdraw to conceal their bodies, or lack time for interpersonal connections due to the demands of disordered eating behaviors. Severe illness may also elicit rejection or misunderstanding from others, further reinforcing loneliness. These processes can create a self-perpetuating cycle in which social withdrawal facilitates eating disorder behaviors, while the physiological effects of starvation, restriction, purging, and low weight exacerbate emotional dysregulation [35]. Importantly, recent evidence highlights that experiences of social exclusion or overinclusion evoke maladaptive emotional and cognitive responses in individuals with eating disorders, suggesting that interpersonal difficulties both maintain and are reinforced by core symptoms [36]. Similarly, maladaptive schemas linked to social situations may amplify vulnerability to perceived rejection and contribute to the entrenchment of loneliness and dysregulation [37]. Taken together, this evidence supports the view that loneliness in eating disorders reflects not only a predisposing vulnerability but also illness-related consequences that interact with emotional functioning in a bidirectional manner, further amplifying dysregulation and perpetuating symptom escalation [38, 39].

The intermediate cluster displayed moderate emotional intelligence and loneliness and included both clinical and non-clinical individuals. This configuration may reflect subclinical vulnerabilities or compensatory mechanisms that buffer the full expression of ED pathology. Notably, this cluster still exhibited significant symptom levels, supporting dimensional models of psychopathology in which intermediate traits confer clinical risk even in the absence of overt dysfunction [40–42]. Longitudinal studies have shown that difficulties in emotion regulation and heightened loneliness prospectively predict the onset and maintenance of disordered eating behaviors [43–45]. On this basis, individuals within such intermediate profiles may be considered at heightened risk, and preventive interventions that strengthen emotional competence and reduce social isolation could be valuable before more entrenched pathology develops [46, 47]. However, given the cross-sectional nature of the present study, these implications remain tentative and require confirmation in longitudinal and interventional research.

Conversely, the most adaptive profile, characterized by high emotional intelligence across all domains and low loneliness, included mostly healthy controls along with ED participants presenting with relatively less severe symptomatology. Rather than reflecting diagnostic subtypes, this pattern highlights a subgroup whose emotional and interpersonal resources may mitigate the burden of illness. Such resilience factors could explain why some individuals maintain lower levels of psychopathology despite exposure to ED risk, in line with evidence that emotional intelligence protects against diverse psychological symptoms and promotes more adaptive social functioning [13, 14, 48, 49].

Emotional profiles in our sample showed only partial overlap with diagnostic categories, supporting the view that transdiagnostic and dimensional approaches may better capture individual variability in EDs [50–52]. The presence of high-functioning emotional profiles among some ED cases indicates that emotional functioning may vary independently of symptom type or illness duration, which could have implications for treatment planning. Regression analyses identified self-emotion appraisal and regulation as significant predictors of clinical status, over and above loneliness and demographic factors, underscoring the potential clinical importance of intrapersonal competencies. However, given the cross-sectional design, it is not possible to determine whether lower emotional intelligence is a vulnerability factor, a consequence of illness, or both. Prior findings suggest that emotional dysfunction often persists beyond improvements in behavioral symptoms [53], pointing to the need for interventions that specifically address these processes. Finally, the lack of differences in emotional profiles by illness duration or hospitalization history suggests that these traits may represent relatively stable characteristics, though longitudinal research is needed to confirm this.

### Clinical implications

These findings hold several clinically relevant implications, though they must be interpreted within the limits of a cross-sectional design.

First, assessing emotional intelligence and perceived loneliness may offer meaningful, transdiagnostic markers for identifying individuals at elevated risk of severe eating disorder psychopathology. Unlike symptom-based screening tools, emotional and interpersonal traits may capture underlying vulnerabilities that cut across diagnostic categories. However, longitudinal research is required to determine whether these markers reliably predict illness onset, maintenance, or relapse. Second, the identification of distinct emotional-loneliness profiles suggests the need for tailored interventions. While evidence-based approaches such as CBT-E remain the gold standard, residual emotional dysregulation and loneliness at treatment completion have been linked to poorer outcomes and higher relapse risk in some studies [45, 54, 55]. Therefore, it may be valuable to complement standard treatments with additional modules focused on emotional awareness, interpersonal connectedness, and regulation skills—particularly when these difficulties persist and the patient identifies them as ongoing concerns. In addition to well-established treatments such as emotion-focused therapy (EFT), dialectical behavior therapy (DBT), and mentalization-based therapy, targeted programs like Cognitive Remediation and Emotion Skills Training (CREST) have shown promise in improving emotional processing in individuals with anorexia nervosa and related presentations [56–58]. At present, however, evidence for tailoring interventions specifically based on these profiles is preliminary, and longitudinal studies are required to determine whether such adaptations improve outcomes beyond established treatments.

Third, the emergence of these profiles irrespective of diagnosis supports the integration of emotion- and relationship-focused assessments into early detection and prevention frameworks [34, 59]. Screening for emotional processing difficulties could enhance the identification of individuals at risk before the onset of full-blown eating disorder symptomatology, though prospective validation is needed.Finally, future clinical research should explore how early relational experiences—such as attachment insecurity or childhood trauma—interact with emotional intelligence and loneliness to shape developmental pathways to disordered eating [22, 60]. Longitudinal and multimethod approaches (e.g., behavioral, physiological, neurocognitive) will be crucial to establish causal mechanisms and to refine personalized, developmentally informed treatment planning.

### Limitations and future directions

Despite its strengths, this study has limitations. Its cross-sectional design precludes causal inferences. All measures were based on self-report, potentially introducing bias or inaccuracies in emotional self-assessment. The sample consisted exclusively of White, Italian, cisgender women, and no data were collected on sexual orientation or other equity-seeking groups (e.g., racial/ethnic minorities, gender-diverse individuals, socioeconomically disadvantaged populations). These characteristics limit the generalizability of the findings. In addition, individuals with ARFID were excluded because the EDE-Q does not adequately capture ARFID-specific symptoms, leaving uncertain whether the identified emotional–loneliness profiles extend to this population. Future research should explore these emotional profiles longitudinally, examine gender and cultural moderators, and integrate multimodal assessments (e.g., behavioral, physiological, neurocognitive) to capture emotional processes with greater precision.

## Conclusion

This study underscores the importance of emotional intelligence and loneliness as transdiagnostic markers that cut across traditional eating disorder diagnoses. The identification of distinct emotional–loneliness profiles suggests that these traits may be closely linked with illness severity and persistence, beyond diagnostic category alone.

The co-occurrence of low emotional intelligence and high loneliness with greater symptom severity highlights the potential clinical relevance of targeting emotional and interpersonal functioning. However, given the cross-sectional nature of the data, further longitudinal and treatment-focused research is needed before confirming these constructs as core therapeutic targets. Conversely, the presence of more adaptive emotional profiles among some individuals with eating disorders points to resilience factors that may help guide individualized treatment approaches.

By moving beyond categorical diagnoses and focusing on underlying emotional traits, these results support a more dimensional, personalized approach to eating disorder care. Future research should further explore how interventions that enhance emotional intelligence and reduce loneliness can improve outcomes across diagnostic boundaries.

## Data Availability

Data supporting the findings of this study are available from the corresponding author, upon reasonable request.
